# Editorial: Oral immunology – interplay of molecules, cells and oral tissue environment

**DOI:** 10.3389/fimmu.2025.1729801

**Published:** 2025-11-10

**Authors:** James Deschner, Kerstin Galler, Jonathan Jantsch

**Affiliations:** 1 Department of Periodontology and Operative Dentistry, University Medical Center of the Johannes Gutenberg University, Mainz, Germany; 2 Department of Operative Dentistry and Periodontology, Friedrich-Alexander-Universität Erlangen-Nürnberg (FAU), Erlangen, Germany; 3 Institute for Medical Microbiology, Immunology, and Hygiene, University Hospital Cologne and Faculty of Medicine, University of Cologne, Cologne, Germany

**Keywords:** immunology, oral, periodontal, dental, orthodontic, maxillofacial, microenvironment, immunology & inflammation

The complex interplay between tissue, the microbiome, and the immune system is crucial to our understanding of health and disease (1–3). In the oral cavity in particular, tissues such as gingiva and mucosa are constantly challenged by myriads of bacteria that form the oral microbiome. However, in dentistry, these molecular and cellular interactions have not been explored in detail so far (4). Consequently, immunological approaches to diagnosing and treating oral diseases have not yet been widely adopted, particularly in the fields of periodontology, endodontology, orthodontics and maxillofacial diseases (5). This Research Topic aimed to improve our understanding of these oroimmunological processes in the three oral domains, pertaining to periodontal, dental, and maxillofacial diseases, in order to advance our understanding of oral immunity in health and disease. This Research Topic of interdisciplinary articles highlights the importance of immunological research in dentistry, with the aim of paving the way for new preventive, diagnostic and therapeutic strategies for all oral diseases.

Schröder et al. studied the role of the myeloid p38α/MAPK signaling pathway in orthodontic tooth movement under the influence of different dietary salt concentrations. High salt intake increased NFAT5 and prostaglandin endoperoxide synthase-2 expression as well as tooth movement only in control animals, but not in *p38α*^Δmyel^ mice. The results show that myeloid p38α/MAPK is crucial for the salt-dependent promotion of tooth movement.

While this article investigated the role of signaling pathways in myeloid cells in salt-triggered orthodontic tooth movement, the work of Fan et al. sheds light on molecular mechanisms of accelerated tooth movement promoted by corticotomy. The single-cell RNA sequencing analysis revealed upregulation of iron metabolism-related genes in macrophages in alveolar bone after the corticotomy. The transcription factor Atf3 was linked to a new macrophage state that interacts closely with osteoclasts and might represent a potential therapeutic target. These findings on immune cell function in tooth movement demonstrate the importance of the interaction between immune cells and tissues.

This concept is also relevant to the development of oral diseases such as cancer, as demonstrated by Trumet et al. using periodontitis and oral squamous cell carcinoma (OSCC) as examples. The authors used radiographic bone loss as a proxy for periodontitis and corrected for confounding factors. In the OSCC group, the bone loss was significantly more pronounced. These data provide evidence of an association between periodontitis-derived inflammation and the malignant transformation of oral epithelium.

In a follow-up study, Trumet et al. analyzed the specific composition of immune cells in precancerous and malignant lesions. They compared the immune cell composition in oral leukoplakia, lichen planus, periodontitis, and OSCC by tissue microarray in combination with 4-plex immunofluorescence. It revealed increased IL-23R expression, macrophage infiltration, as well as M2-like macrophage polarization in leukoplakia and lichen planus compared to controls. The metastatic OSCC had more macrophages than non-metastatic OSCC. The results suggest involvement of macrophages and IL-23 signaling pathways in oral carcinogenesis.

The complex immunological processes involved in oral diseases are also influenced by systemic diseases such as rheumatoid arthritis. The study by Andreev et al. investigated temporomandibular joint (TMJ) involvement in rheumatoid arthritis using a human TNF transgenic mouse model. The animals demonstrated typical arthritis features, such as synovial inflammation, bone resorption, and increased osteoclast numbers. Compared to the ankle joint, the TMJ exhibited a unique pattern of immune cell infiltration and cytokine expression. The RNA sequencing revealed an increased expression of genes associated with energy consumption and bone resorption-related enzymes in TMJ, underlining its particular vulnerability in arthritis.

In addition to joint tissues, the dental pulp is also a key site of immunological activity, as Pohl et al. describe in their review of the molecular and cellular mechanisms of pulp inflammation. The dental pulp is a unique tissue within each tooth that can develop painful inflammation (pulpitis) in the event of microbial infections or injuries. Complex immune responses are established to combat pathogens and promote new dentin formation at the site of infection. The interaction of immune and non-immune cells via cytokines, chemokines, and other mediators leads to tissue reactions and structural changes in the pulp that can become irreversible when a certain threshold value is exceeded. A better understanding of the cellular and molecular mechanisms in dental pulp is crucial.

Likewise, complex immune processes have been observed in inflamed periodontal tissues, as outlined in the overview by Neurath and Kesting. Microorganisms, their components, and products activate both the innate and adaptive immune systems, leading to the synthesis of proinflammatory mediators. In the experimental models of periodontitis, TNF and the IL-23/IL-17 axis play a decisive role in this disease. Such and other molecules not only mediate periodontal inflammation and destruction, but hold great potential to serve as markers for improved periodontal diagnosis and therapy in the future.

The oral microbiome consists not only of the dental/periodontal microbiome, but also of the tongue microbiome. Numerous studies show that periodontal disease is also associated with kidney disease. The study by Hoefer et al. investigated whether an intensive oral prophylaxis program influences the tongue microbiome in children with chronic kidney disease compared to standard prophylaxis. No significant differences in bacterial diversity or composition were found between the two treatment groups over a six-month period. Despite reduced signs of inflammation, the tongue microbiome remained stable, underscoring its resilience even in patients with kidney disease.

Although the stability of the microbiome indicates that clinical interventions may exert only subtle effects, López-Valverde et al.'s findings suggest that new therapeutic approaches could positively impact the inflammatory environment around implants. This systematic review and meta-analysis focused on the efficacy of probiotics in peri-implant diseases. The authors found a tendency towards a positive effect of probiotics on inflammatory markers and clinical values in both peri-implant diseases, suggesting a potential benefit of probiotics as an adjunctive therapy. However, further research is needed in this field.

Overall, this Research Topic demonstrates that the interactions between molecules, cells and tissue microenvironments are complex, and that immunoinflammatory processes influence the clinical manifestation, diagnosis and treatment of oral diseases. Further preclinical and clinical studies and interdisciplinary collaboration are required to unravel these highly complex, dynamic interactions.
